# Ultrasonography of the Vagus Nerve in Parkinson's Disease

**DOI:** 10.3389/fneur.2018.00525

**Published:** 2018-07-02

**Authors:** Nadin Fedtke, Otto W. Witte, Tino Prell

**Affiliations:** ^1^Department of Neurology, Jena University Hospital, Jena, Germany; ^2^Center for Healthy Ageing, Jena University Hospital, Jena, Germany

**Keywords:** vagus nerve, sonography, cross-sectinoal area, Parkinson's disease, polyneuropathy, bradykinesia, dysautonomia

## Abstract

Based on the topographic distribution of α-synuclein-enriched Lewy bodies, it has been hypothesized that Parkinson's disease may start in the gastrointestinal tract and gain access to the central nervous system via the vagus nerve. Since ultrasonography is a powerful tool to study peripheral nerve disturbances, we conducted ultrasonography of the vagus nerve in 32 patients with Parkinson's disease, 15 disease controls and 15 healthy controls. The cross-sectional area and echogenicity measured on transverse scans of the vagus nerve did not differ significantly between these groups. Therefore, the observed intraneuronal changes in Parkinson's disease are not associated with ultrasonographic disruptions of the vagus nerve integrity.

**HIGHLIGHTS**

- We studied ultrasonography of the vagus nerve in 32 patients with Parkinson's disease and in 15 disease controls and 15 healthy controls.
- The sonographic cross-sectional area measured using high-frequency linear array transducers did not differ significantly between both groups.
- Ultrasonography of the vagal nerve does not reflect cellular damage caused by α-synuclein-enriched Lewy bodies in nerves of patients with Parkinson's disease.

## Introduction

It has been hypothesized that neuropathological process that lead to Parkinson's disease may start in the enteric nervous system and spread rostrocranial via the vagus nerve to the substantia nigra (1–3). This gut-brain transmission scenario is controversially discussed within the movement disorders community (4). Nevertheless, the pathological α-synuclein signature can be observed in the peripheral nervous system in patients with Parkinson's disease (5).

Since high-frequency ultrasonography provides a valuable tool to study both peripheral nerves and the vagus nerve and is increasingly being used in the diagnosis of polyneuropathies (6), we aimed to answer the question whether patients with Parkinson's disease have sonographic abnormalities in the vagus nerve.

## Methods

The study was approved by the local ethics committee and has been performed in accordance with the ethical standards laid down in the 1964 Declaration of Helsinki and its later amendments. All patients gave their written informed consent. We performed standardized ultrasonography of the vagus nerve at the lateral margins of the anterior cervical region beneath the sternocleidomastoid muscle in 20 male and 12 female patients with Parkinson's disease, 15 age-and sex-matched disease controls (6 stroke, 7 essential tremor, 2 headache) and 15 age-and sex-matched healthy controls (15 MHz, Toshiba Aplio 400). The nerve cross-sectional area (CSA) was determined by tracing the nerve area within the hyperechoic epineurium. Polyneuropathy was ruled out by means of additional nerve conduction studies and ultrasonography of the median nerve, tibial nerve and suralis nerve as previously described (7).

The Movement Disorder Society-sponsored revision of the Unified Parkinson's Disease Rating Scale (MDS-UPDRS I–IV) and Hoehn and Yahr staging were used to evaluate motor and non-motor symptoms. The distinct PD subtypes (postural instability and gait difficulty-predominant type, indeterminate/mixed type and tremor-type) and bradykinesia score were calculated from the MDS-UPDRS as previously described (9, 10).

The SPSS statistical computer package (version 23.0; IBM Corporation, Armonk, NY, USA) was used for all statistical analyses. All continuous data are presented as mean ± standard deviation. Categorical variables are presented as percentages (%). Prior to statistical analysis, data were checked for outliers and for normality using the Shapiro-Wilk's test. For comparisons of CSA between the three groups an ANOVA with Bonferroni correction was used. *T*-test was used to compare patients with and without constipation. Pearson correlation was used for normally distributed values. Statistical significance was set at *P* < 0.05.

## Results

**Figure 1:**
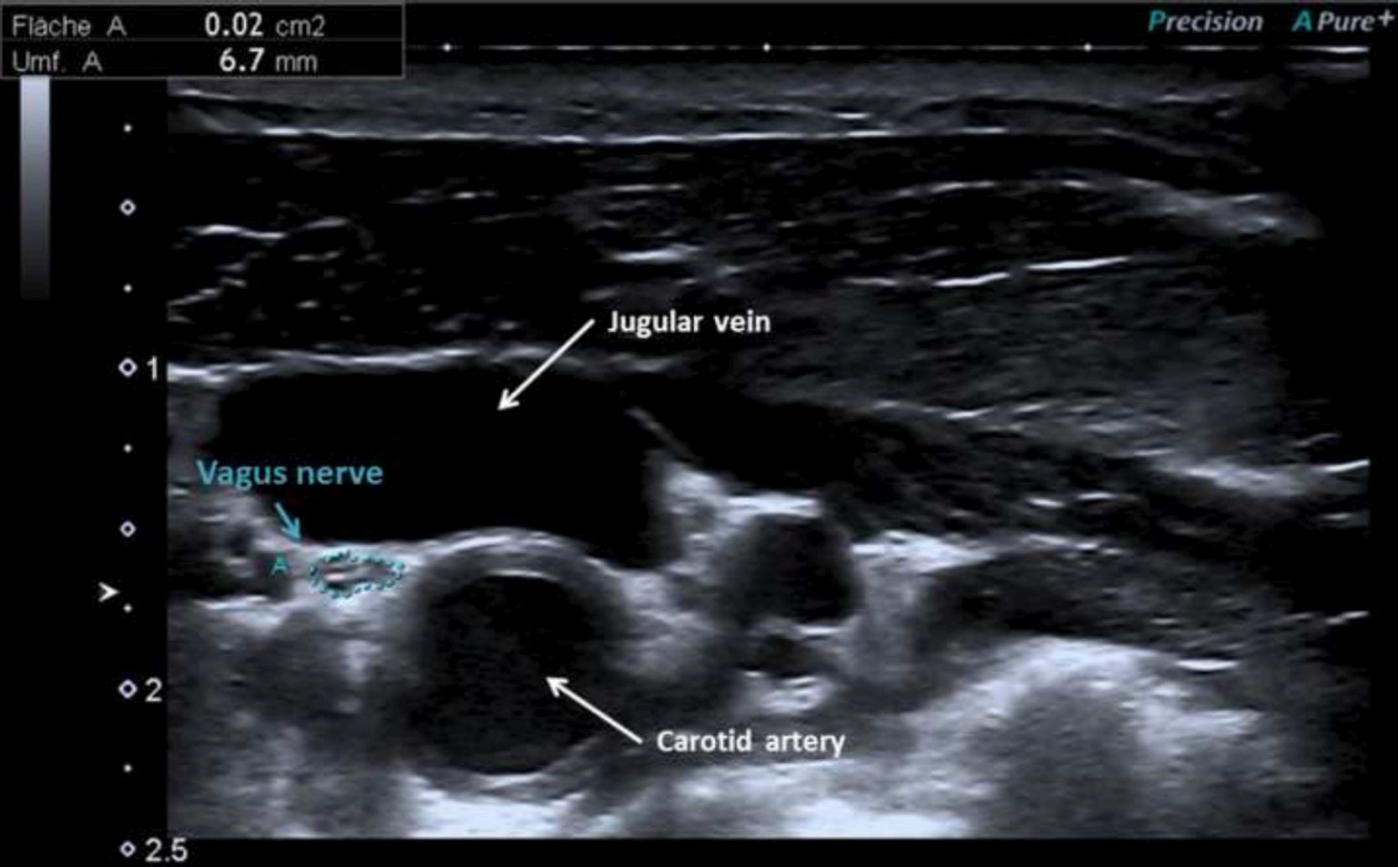
Vagus nerve at the lateral margins of the anterior cervical region beneath the sternocleidomastoid in a patient with Parkinson's disease with cross-sectional area of 2 mm^2^.

Patients with Parkinson's disease were characterized by a mean age of 70 ± 6 years and moderate motor impairment (mean MDS*-*UPDRS III = 33 ± 12, mean UPDRS II = 17 ± 7). Of note, 3.1% of patients were mildly, 62.5% moderately and 34.4% severely affected (Hoehn and Yahr stage I, II-III, IV respectively). The right CSA of the vagal nerve was significantly larger than the left (*P* = 0.021). The CSA (mm^2^) measured on transverse scans of the vagal nerve did not differ significantly between healthy controls (right = 2.7 ± 0.7, left = 2.4 ± 0.7), disease controls (right = 2.7 ± 0.6, left = 2.3 ± 0.4) and patients with Parkinson's disease (right = 2.9 ± 0.7, left = 2.6 ± 0.7) (ANOVA, Bonferroni correction, *P* > 0.05). Figure 1 shows an ultrasonographic example of the vagus nerve in a patient with Parkinson's disease. In addition, nerve echogenicity, like the fascicle appearance, did not differ between the three groups. CSAs of the vagal nerves did not significantly correlate with disease duration, Hoehn and Yahr stage, and the tremor/PIGD index. The CSAs of the vagal nerve did not significantly differ between patients with and without constipation (MDS-UPDRS item 1.11). However, the CSA of right vagus nerve was significantly positively correlated with the bradykinesia score (right Pearson's *r* = 0.537, *P* = 0.003; left Pearson's *r* = 0.348, *P* = 0.069).

There was no significant difference of the CSA in the median, tibial and suralis nerves between patients with Parkinson's disease and controls (anova, Bonferroni correction, *P* > 0.05).

## Discussion

Ultrasonography of the vagus nerve can support the diagnosis of several neuropathies. A slightly reduced CSA of the vagus nerve was observed in patients with diabetes mellitus compared to healthy controls and this was interpreted as vagal atrophy or degeneration (11). In contrast, vagal hypertrophy or focal enlargement can occur in immune-mediated or vasculitic neuropathies (8, 12). However, in patients with Parkinson's disease, high-resolution ultrasonography did not reveal detectable structural disturbances in the vagal nerve in comparison to healthy controls. According to the known reference values the right vagus nerve was larger than the left one (13). The CSA of the right vagal nerve correlated with the bradykinesia assessed by the MDS-UPDRS III. Indeed, the bradykinesia-dominant subtypes seem to be associated with widespread Lewy body pathology in the sympathetic central nervous system (14, 15). However, this correlation cannot be fully explained and must be interpreted with caution because of the absence of the difference to controls and the natural variability of nerve size.

The study has several limitations. We used state of the art high-resolution ultrasonography but we cannot rule out that a higher-resolution would be able to detect structural disturbances in the vagal nerve of patients with Parkinson's disease. Nevertheless, our study showed that there is no prominent nerve enlargement as known from immune-mediated neuropathies. For example, Grimm et al. (12) observed a focal swelling of both vagal nerves of up to 9 mm^2^ CSA in the left and 6 mm^2^ CSA in the right. In comparison, as in our study, the normal area size lies between 2 and 3 mm^2^ (7, 8, 13).

In conclusion the sonographic structure of the vagus nerve is unremarkable and ultrasonography of the vagus nerve seems to have no potential as biomarker in Parkinson's disease.

## Author contributions

NF and TP: design of the study; NF and TP: acquisition and analysis of data; NF and TP: drafting; OW: revising work critically for important intellectual content.

### Conflict of interest statement

The authors declare that the research was conducted in the absence of any commercial or financial relationships that could be construed as a potential conflict of interest.
